# Retinal neurodegeneration and synaptic loss in relations to core‐proteinopathies and cognitive scores in MCI and AD patients

**DOI:** 10.1002/alz.087799

**Published:** 2025-01-03

**Authors:** Edward K Robinson, Altan Rentsendorj, Dieu‐Trang Fuchs, Jean‐Philippe Vit, Bhakta Prasad Gaire, Miyah R Davis, Ousman Jallow, Lon S. S. Schneider, Debra Hawes, Keith L Black, Yosef Koronyo, Maya Koronyo‐Hamaoui

**Affiliations:** ^1^ Neurosurgery Department, Cedars‐Sinai Medical Center, Los Angeles, CA USA; ^2^ Department of Neurosurgery, Maxine Dunitz Neurosurgical Research Institute, Cedars‐Sinai Medical Center, Los Angeles, CA USA; ^3^ Cedar Sinai Medical center, Los Angeles, CA USA; ^4^ Banner Alzheimer’s Institute, Phoenix, AZ USA; ^5^ Keck School of Medicine of USC, Los Angeles, CA USA; ^6^ Department of Psychiatry and Behavioral Sciences, Keck School of Medicine, University of Southern California, Los Angeles, CA USA; ^7^ Alzheimer’s Disease Research Center, Keck School of Medicine, University of Southern California, Los Angeles, CA USA; ^8^ University of Southern California, Los Angeles, CA USA; ^9^ Department of Pathology, Keck School of Medicine, University of Southern California, Los Angeles, CA USA; ^10^ Department of Neurology, Cedars‐Sinai Medical Center, Los Angeles, CA USA; ^11^ Department of Biomedical Sciences, Division of Applied Cell Biology and Physiology, Cedars‐Sinai Medical Center, Los Angeles, CA USA

## Abstract

**Background:**

Alzheimer’s disease (AD) is the foremost cause of global dementia, also characterized by retinal changes involving Aβ, hyperphosphorylated‐tau (p‐tau), neuronal degeneration, and tissue atrophy. Mitochondrial‐driven reactive oxygen species (ROS) production, linked to synaptic dysfunction, is common to various neurodegenerative conditions, including AD. Despite synaptic dysfunction being an early predictor of cognitive decline in AD, its occurrence in the AD retina is unexplored. This study examines retinal neurodegeneration and synaptic integrity in correlation with AD brain and retinal pathology.

**Method:**

Utilizing histological and mass spectrometry approaches, we assessed atrophy, early apoptosis, cell death markers, ROS levels and pre‐ and post‐synaptic markers in post‐mortem retinas from confirmed AD and mild cognitively impairment (MCI) patients compared to age‐ and sex‐matched subjects with normal cognition (NC). We applied Pearson’s (*r*) correlation analyses to determine the relationships between retinal tauopathy, amyloidosis, gliosis, ROS, apoptosis, and tissue atrophy severity, and their association with synaptic biomarkers. These retinal observations were then correlated with AD‐related brain parameters and cognitive scores.

**Result:**

Proteomics analysis in AD patients’ retinas revealed a strong link between synaptic loss and neurodegeneration. In MCI and AD, we detected highly significant neurodegeneration (Nissl staining) in the outer nuclear layer (ONL; 28%; 23%), inner nuclear layer (INL; 38%; 21%), and ganglion cell layer (GCL; 54%; 52%), accompanied by increased ROS levels (1.9; 2.2‐fold; by DHE staining) respectively, compared to NC. Marked pre‐ (Synaptophysin: 56%; 77%) and post‐ (PSD95: 44%; 66%) synaptic losses were detected in the inner and outer plexiform layers of MCI and AD patients, along with strong negative correlations with accumulation of retinal pSer396‐tau, Aβ_42_ and gliosis. The retinas of these patients also exhibited significant increases in early apoptotic markers. Remarkably, retinal synaptic loss and neurodegeneration had a strong negative correlation with severity of cognitive impairment, BRAAK stage, and ABC score.

**Conclusion:**

Our study reveals pronounced retinal neurodegeneration at pSer396‐tau and Aβ_42_ accumulation sites, coupled with increased retinal oxidative stress in MCI and AD patients. Elevated retinal ROS and pre‐apoptotic cells may contribute to synaptic loss and neuronal death. These findings suggest that retinal synaptic loss and neurodegeneration may predict AD status.

